# Psychosocial factors underlying physical activity

**DOI:** 10.1186/1479-5868-4-38

**Published:** 2007-09-19

**Authors:** Juan Zhang, Susan E Middlestadt, Cheng-Ye Ji

**Affiliations:** 1 Department of Applied Health Science, Indiana University, Bloomington, Indiana, USA; 2 School of Public Health, Peking University and National Institute for Child and Adolescent 38^# ^Xueyuan Road, Haidian District, Beijing, P.R. China

## Abstract

**Background:**

Given the increasing importance of obesity in China, prevention interventions encouraging physical activity by middle school students are needed. The purpose of this study is to illustrate how a rapid elicitation method can be used to identify salient consequences, referents, and circumstances about physical activity as perceived by middle school students and to provide suggestions for interventions and quantitative research.

**Method:**

A theory-based qualitative study using a self-completion elicitation was conducted with 155 students from two middle schools in Beijing, China. Following the Theory of Planned Behavior, six open-ended questions asked students for their perceptions about performing physical activity at least 60 minutes each day: advantages of participating in physical activity; disadvantages of doing so; people who approve of participation; people who disapprove; things that make it easy; and things that make it hard. Content analysis revealed categories of salient consequences, reference groups, and circumstances.

**Results:**

While the three most frequently mentioned advantages elicited from the students were physical health consequences (e.g., will strengthen my body (58.7%)), four of the salient advantages were not (e.g., will improve my grades (12.2%)). Parents were the most frequently mentioned social referent (42.6% as approving; 27.7% as disapproving) when students were asked who might approve or disapprove of their participation. Circumstances perceived to hinder daily physical activity included having too many assignments and not having enough time.

**Conclusion:**

While many of the beliefs about physical activity elicited from this study were similar to those found with students from England and the US, several were unique to these students from Beijing. The results of this qualitative research suggest that interventions to encourage physical activity among middle school students should address: perceived consequences of physical activity on academic achievement and other factors beyond physical health; barriers of not having enough time and having too many assignments perceived to hinder frequent physical activity; and parental approval. More rigorous research on psychosocial determinants with close-ended items developed from these open-ended data and with larger sample sizes of students is necessary. Research with parents and school staff will be needed to understand the perceptions of these stakeholder groups key to creating the students' social environment.

## Background

Overweight and obesity are becoming important public health problems in China among both adults and youth. A nationally representative cross-sectional study reported the age-standardized prevalence of overweight is 28.9% among Chinese adults [1]. The rate of overweight and obesity observed in a study of urban school children increased from almost 8% in 1991 to more than 12% six years later [2]. The National Student Constitution Survey (NSCS) in 1985, 1991, 1995, and 2000 reported the prevalence of obesity among in-school students has increased alarmingly in the metropolitan areas of China over the past 15 years [3].

It is logical to link the increasing obesity rates in developing countries with a progressive introduction of factors associated with obesity in developed societies, such as sedentary lifestyle and consumption of high fat and fast foods [4]. A cross-sectional study conducted in 9 provinces of China reported that in-school activities and active commuting represent the most important forms of moderate or vigorous physical activity of the Chinese youth [5]. According to the report on Global School-based Student Health Survey, only 21% of Chinese students living in Beijing between 13 and 15 years of age are engaging in physical activities for at least 60 minutes per day outside physical education class. In this study, physical activity was defined as participating in physical activity which increases the heart rate and makes the person get out of breath some of the time; participants were asked not to count time in physical education or gym class [6]. A review of factors influencing obesity in the United States indicated that adequate participation in physical activity in childhood may be of critical importance and that physical activity habits developed early in life may persist into adulthood [7]. Additionally, there is an emerging body of evidence recognizing the association between physical activity and improved academic outcomes as well as the association between physical activity and reduced incidence of depression, anxiety and fatigue [8-13]. In the US, a number of successful prevention programs have focused on middle schools presumably because middle school students are more cognitively mature and have more control over physical activity options than elementary school students [14]. Therefore, to prevent and control the epidemic of childhood obesity in China, effective prevention programs addressing physical activity are needed and middle school is a good place to start.

Experience has shown behavioral interventions based on an empirical understanding of psychosocial factors underlying people's decisions are more likely to be effective. Stated another way, research identifying the factors associated with the target behavior among representatives of the priority group is an important prerequisite to effective interventions. Meta-analytic reviews [15,16] have demonstrated that the Theory of Planned Behavior [17,18] can be used to understand the psychosocial determinants of physical activity behaviors and, therefore, can provide a foundation for intervention design to increase physical activity.

Briefly, the Theory of Planned Behavior (TPB) proposes that the immediate determinants of volitional behavior are a person's intention to engage in the behavior and perceived behavioral control [17,18]. Intention is, in turn, determined by a weighted combination of attitude towards the act, subjective norm, and perceived behavioral control. And, finally, attitude towards the act, subjective norm, and perceived behavioral control are determined by sets of salient behavioral, normative, and control beliefs. According to the TPB, only salient beliefs operate as psychosocial determinants. Therefore, a critical step in the application of the TPB is a salient belief elicitation study of the target behavior with members of the population of interest [15]. The purpose of an elicitation study is to determine the top-of-the-mind or salient consequences, referents, and circumstances that form the belief structure underlying the intention. It is essential that these salient beliefs be elicited for each new behavior and for each new priority group. To determine the modal salient beliefs, Ajzen and Fishbein [17,18] recommend that researchers: (a) conduct an elicitation study with representatives of the priority group using open-ended questions to identify salient consequences, referents, and circumstances; (b) perform a content analysis to rank-order the beliefs; and (c) select the 5 to 10 most frequently mentioned items as the salient set.

While the TPB has been supported in a number of domains, including physical activity, and the TPB clearly calls for a salient belief elicitation, additional attention to the belief elicitation step is warranted. The explanatory power of the TPB may be compromised by conceptual and methodological concerns [19] and a main methodological concern for understanding exercise intention and behavior is the limited use of elicitation studies [20]. In fact, a recent review of 150 TPB studies of physical activity showed that only 47 of these studies had conducted an elicitation when applying the theory. Several recent studies have focused explicitly on the elicitation method. Two studies have examined the effect of question order and question wording [21,22]; another study compared individual elicited to modal salient normative beliefs [23]. And, some studies conducted to support intervention design have focused on the results of the elicitation phase [24,25].

While there is considerable evidence that the Theory of Planned Behavior can be used to understand a variety of behaviors, including physical activity, there is less evidence as to the psychosocial factors underlying physical activity among middle school students. Only two of the TPB studies that elicited salient beliefs were conducted with middle school students and none identified the beliefs among Chinese [20]. A study in England of adolescents between 9 and 11 years of age reported salient consequences (i.e., feel healthy and better; fun and enjoyment; make friends; increased risk of injury; and too much effort) as well as social referents (i.e., parents; grandparents; other family members; friends; and teachers) [26]. A study of US adolescents between 14 and 19 years of age found several consequences (i.e., stay in shape; feel healthy; good about self; control weight and diet; increase energy; tired; and pain), normative beliefs (i.e., parents, siblings, friends, coach, and teacher), and control beliefs (i.e., lacking time; money; support; motivation; exercise facts; and access to equipment or activity programs) [27]. Motl and colleagues identified factors in study of American students based on social cognitive theory (i.e., cope with stress; fun; make new friends; keep in good shape; be more attractive; give me more energy; make me better in other sports; and make me hot and sweaty) [28].

To apply TPB to understand the psychosocial determinants of regular physical activity behavior among the Chinese middle school students, qualitative research is a necessary first step. The purpose of this study is to identify the salient beliefs underlying the decision to participate in physical activity held by this particular priority group of middle school students. Specifically, the study will identify the salient consequences of the behavior, the salient referents or social groups, and the salient circumstances or conditions of participating in at least 60 minutes of physical activity every day as perceived by middle schools students living in Beijing, China. The results of this rapid elicitation method can suggest features of interventions as well as provide input to the design of close-ended items for larger scale theory-based quantitative studies.

## Methods

A qualitative salient belief elicitation study was conducted as a part of cooperative activities of the Collaborative between Peking University Health Science Center and Indiana University. The primary data collection was approved by the Human Subject Committee at Peking University Health Science Center. The National Institute of Children and Adolescent Health of the Peking University facilitated access to schools through its network with the school systems. Researchers from the Department of Applied Health Science of Indiana University conducted the data analysis. The analysis of the existing data was approved by the Human Subject Committee at Indiana University.

### Participants

The 155 study participants were students selected from four classes of two middle schools. In Beijing, middle school or junior high school consists of three grades: Grade 1, 2 and 3. Middle school students are from 13 to 15 years of age. Typically, three, 45-minute physical education classes are offered per week. The classes were selected following a purposive sampling plan by selecting one district in Beijing, two schools within the district, one grade within the school, and two classes within each grade. All students in the selected classes were asked to participate in the study.

More specifically, the research team contacted the School Health administrative agency of Dongcheng district, a district that is average among districts in Beijing in terms of academic grades excluding physical education for their support for this study. The research team contacted administrators from the middle schools in this district to identify study sites. Most schools in the district were not offering physical education courses during the final weeks of the semester when the data were collected. However, it was possible to identify two schools that were offering physical education classes at the time the data were collected and that were willing to participate. To facilitate ease of administration, it was decided to select two classes from Grade 1 in one school and two classes from Grade 2 in the second school. There were six Grade 1 classes in one school and eight Grade 2 classes in the second school. Doctors working in the respective school clinics were used as key informants to classify the classes in each grade into two broad groups (high and low) with respect to the average academic grades (physical education class excluded) in the grade of interest. One class ranking high and one class ranking low was selected from each school. All the students from each of the four classes were invited to participate in the study; all students who were asked agreed to participate in this study.

### Instrument

The behavior selected for study was "participating in at least 60 minutes of physical activity every day." Following the WHO definition [29], physical activity was defined as "any activity that increases your heart rate and makes you get out of breath some of the time" and physical activity in physical education class was excluded. The semi-structured self-completion questionnaire began with a cover sheet that described the purpose of the study. Next came close-ended questions that were derived from the Global School-based Health Survey [29] and that assessed demographic characteristics, height, and weight, level of physical activity and inactivity, and information about physical education classes. Finally, several open-ended questions (Table 1) were used to identify salient consequences, social referents, and circumstances of participating in physical activity.

**Table 1:** Open-Ended Questions

**Constructs**	**Questions**
Salient Advantages	What do you see as the advantages or good things that would happen if you participate in physical activity at least 60 minutes per day?
Salient Disadvantages	What do you see as the disadvantages or bad things that would happen if you participate in physical activity at least 60 minutes per day?
Salient Referents Who Approve	Who do you think would agree or approve if you participate in physical activity at least 60 minutes per day?
Salient Referents Who Disapprove	Who do you think would object or disapprove if you participate in physical activity at least 60 minutes per day?
Salient Easy Circumstances	What makes it easier for you to participate in physical activity at least 60 minutes per day?
Salient Hard Circumstances	What makes it difficult or impossible for you to participate in physical activity at least 60 minutes per day?

### Procedure

The self-completion instrument was administered during physical education class by two members of the research team who were not associated with the school. Neither the classroom teacher nor the physical education teacher was present during data collection. The facilitators introduced the purpose and value of the study. They emphasized that data collection was anonymous; that there were no right or wrong answers; and that participation was completely voluntary and would have no effect on their grades. The semi-structured questionnaire was completed within 30 minutes. Simple probing was used with all the students, including, for example, "who would care that you always do physical activity every day," "who would disagree with you doing physical activity," and "what are good things if you do physical activity everyday." A group discussion was conducted at the end to get feedback on the students' view of the method.

### Content analysis

The purpose of the content analysis was to identify categories of positive and negative outcomes or consequences of performing the behavior, of individuals or social groups who serve as social referents, and of easy and difficult circumstances to perform the behavior. Therefore the content analysis was conducted by two researchers who were familiar with the Theory of Planned Behavior and who could abstract the theoretical constructs from the responses. One of the coders was also a native speaker of Mandarin studying for a PhD in Health Behavior in the United States. The responses to open-ended questions were translated from Chinese to English by the bilingual researcher and then entered verbatim into a word processing file. Similar responses were grouped together to form major categories of responses for each question. These groups of responses were reviewed by two researchers to create a final set of coding categories and to phrase the categories in terms of the appropriate theoretical construct. The bilingual researcher checked with the initial responses in Chinese to ensure cultural and linguistic accuracy. The data were coded into these categories and entered into an SPSS file.

### Descriptive analysis

The file of open-ended data was integrated with the file of close-ended data. Students who indicated they had participated in at least 60 minutes of physical activity for four or more days in the past week were classified as doers; those who reported three or few days were classified as non-doers. Cross tabulation analyses were conducted to compare the percentage mentioning each category of salient consequences, social referents, and circumstances separately among doers and non-doers. Chi square analyses revealed no significant differences between doers and non-doers. Therefore the results are presented as the percent mentioning each category of responses over the entire sample of students.

## Results

### Description of participants

Almost all of the participants (98%) were between 13 and 15 years of age; about half were male and about half were female (Table 2). Thus, the sample was similar to middle school students in Beijing and China with respect to age and gender [6]. Only 14.1% of the middle school students in this study performed at least 60 minutes of physical activities every single day (Table 3). Approximately 86% of this sample of middle school students watched TV two hours or less per day (Table 3). The mean BMI of students was 19.3 ± 3.7. Almost all the students (97.4%) attended the three physical education classes offered by the school every week.

**Table 2:** Demographic Description of the Study Sample from Close-Ended Questions

Descriptive Information	Percent
Grade	
Grade 1	38%
Grade 2	62%
Age	
Under 13 years old	2%
13 to 15 years old	96%
Over 15 years old	1%
Missing	1%
Gender	
Female	47%
Male	53%

**Table 3:** Description of the Study Sample from Close-Ended Questions

	Percent
Perceived General Health Status	
Very bad	1.3%
Just so so	12.8%
Good	14.1%
Very good	37.8%
Extremely good	31.4%
Days Participate in Physical Activity at Least 60 Minutes During the Past Week	
0 day	15.4%
1 day	13.5%
2 days	11.5%
3 days	20.5%
4 days	11.5%
5 days	7.7%
6 days	5.1%
7 days	14.1%
Hours Viewing TV Per Day	
Don't watch TV	21.2%
Less than 1 hour	21.2%
1 hour per day	21.8%
2 hours per day	21.8%
3 hours per day	4.5%
4 hours per day	5.1%
5 more hours per day	3.8%

### Salient consequences

Tables 4 and 5 present the salient consequences of participating in physical activity perceived by these middle school students. Comparing these tables reveals that these students saw more positive outcomes than negative ones. The most frequently mentioned advantage (i.e., "will strengthen my body") was mentioned by over half (58.7%) of the students. Many of the perceived consequences involved physical health outcomes. In fact, the three most frequently advantages of physical activity (i.e., "will strengthen my body", "will improve health", and "will get more exercise") can be viewed as physical health consequences. However, these middle school students also mentioned outcomes beyond physical health (e.g., "will relax me," "will improve my physical education grades," and "will give me energy to study"). The most frequently mentioned disadvantage, "will take too much time," was mentioned by 40.6% of the students. In addition, 24.5% of the students specifically mentioned that participating in physical activity "will take time away from studies" and 36.8% of the students mentioned that participating in physical activity "will make me tired."

**Table 4:** Salient Advantages of Participating in Physical Activity

Salient Advantage	Percent Mentioning (%)
Will help strengthen my body	58.7
Strengthen body	
Have good body	
Strengthen muscles	
Be tall	
Will improve my health	27.7
Good health	
Do good to heart	
Improve blood circulation	
Strengthen digestive system	
Relax brain	
Strengthen bone and muscles	
Refresh eye	
Will get me exercise	26.5
Do more exercise	
Will relax me	17.4
Have a good body and mind	
Have a good mind	
Relax	
Adjust the mood	
Will strengthen my immunity	15.4
Strengthen the immunity	
Will improve my grades in physical education	12.2
Have a good grades of physical education	
Have better grades in physical education	
Run fast	
Will help me lose weight	9.8
Lose weight	
Burn fat	
Will keep me free from diseases	9.6
Free from diseases	
Free from flu	
Will strengthen my vital capacity	5.8
Strengthen vital capacity	
Increase vital capacity	
Will give me energy to study	5.8
Have energy	
Have strength to study	
Be active or awake in class	
Reduce the academic burden	
Will be fun	5.2
Recreation	
Take full advantage of time	
Have a fruitful life	
Have a lot of fun	
Have more extra activities	
Will keep me in a good shape	3.2
Keep in a shape	
Stay in a shape	
Agility	
Flexibility	

**Table 5:** Salient Disadvantages of Participating in Physical Activity

Salient Disadvantage	Percent Mentioning (%)
Will take too much time	40.6
Waste time	
No time	
Don't have time to watch TV	
Don't have time to play games	
Don't have time to take rest	
Will make me tired	36.8
Feel tired after physical activity,	
Can't recover to normal status	
Will take time away from studying	24.5
Influence study	
Not helpful to study	
Reduce the time to study	
Can't concentrate on study after physical activity	
Don't have good grades	
Will lead to me getting hurt or injured	12.2
Will be injured	
Will feel painful after physical activity	
Will feel sore in the leg	
Will mean having to wash up	3.2
Take a shower	
Need to wash hair	
Need to wash feet	
Need to clean clothes	

### Salient referents

Table 6 shows the individuals and groups mentioned when these students were asked who approved and disapproved of their engaging in physical activity. Clearly, most of the salient referents for this behavior were family members, including parents, mothers, fathers, and grandparents. Teachers and classmates were also mentioned by these middle school students. Doctors and other health professionals were not very frequently mentioned. Parents were the most frequently mentioned approving group and disapproving group and were mentioned more frequently as approving (42.6%) than as disapproving (27.7%). It is interesting to note that when a specific parent was mentioned, mothers were mentioned more frequently than fathers.

**Table 6:** Salient Social Referents for Participating in Physical Activity

Salient Referents	Percent Mentioning as Approving (%)
Parents	42.6
Mother	20.0
Teacher	17.4
Father	15.5
Classmates	4.5
Grandparents	3.9
Doctor	1.3

	Percent Mentioning as Disapproving (%)

Parents	27.7
Teacher	15.4
Mother	12.9
Father	9.0
Grandparents	8.4
Classmates	2.6

### Salient circumstances

Tables 7 and 8 present the circumstances which students believed made participating in physical activity at least 60 minutes every day easier and more difficult, respectively. Comparing the two tables, it can be seen that many of the circumstances (e.g., assignments, time, and weather) were mentioned both as circumstances that make physical activity easy and as circumstances that make it difficult. The most frequently reported facilitator of physical activity, "having fewer assignments", was mentioned by 27.7% of the students. The most frequently mentioned barrier, "having too many assignments", was mentioned by about half (48.4%) of the students. Additionally, "time" was the second most frequently mentioned circumstance; 14.2% of the students mentioned having more time as a facilitator and 18.8% mentioned not having enough time as a barrier. These data also suggest that "having fun activities," "having others to participate with," "approval from others" and "making facilities more available" operate as facilitating and hindering circumstances.

**Table 7:** Salient Circumstances Facilitating Participation in Physical Activity

Salient Circumstances that Facilitate	Percent Mentioning (%)
Having fewer assignments	27.7
Finish the assignment very quick	
Finish the assignment in school	
Don't take extra courses after school	
Have a good academic scores	
Having more time	14.2
Give more time to play computer	
Nothing to do	
Don't see watch	
More time for extra activities	
It is very early when class is over	
Having easy and fun activities to do	12.3
Activity is not too vigorous	
If I go somewhere not far from my home, had better walk or take bicycle	
Activities I am interested in Fun activity	
When I do physical activity, I can listen to music	
Go swimming	
Play basketball or football Contest	
Being on vacation	11.6
Summer vacation	
Winter vacation	
Weekend	
Having good weather	10.3
Nice weather	
Fresh air	
Warm	
Having more PE classes	9.7
The proportion of PE increases	
PE test	
In-school physical activity	
Having approval from my parents and teachers	9.0
Parents approve	
Support from other people	
Encouragement from parents	
School agree	
Senior leaders issue relevant documents	
Having someone go with me	7.7
Go with friends	
Go with classmates	
Parents take me to do exercise	
Go with parents	
Being in a good mood	7.7
Very happy	
Feel very boring	
Bad mood	
Getting rewarded to	5.8
Gain points for exam	
Money	
Can play computer for 120 minutes	
Give me 10 RMB, I will do physical activity	
Encouragement	
Having nothing else to do	2.6
Nothing to do	
Parents don't permit me to play computer	
No good TV program	
Having place or court	2.6
Good court	
Facility is good	
There court in the neighborhood	

**Table 8:** Salient Circumstances Hindering Participation in Physical Activity

Salient Circumstances that Hinder	Percent Mentioning (%)
Having too many assignments	48.4
Busy study schedule	
Too much assignment	
Bad academic scores	
Before exam	
It take long time to work on assignment	
Have too much extra courses on weekend	
Not having enough time	18.8
Time is tight	
No time	
Waste time	
It is very late when class is over	
Have something else to do	
Class teacher taking up the time	
Having bad weather	18.1
Bad weather	
Windy, rainy outside	
Snow, raining	
Air is polluted	
Cold	
Cloudy, raining	
It is hot	
Being tired, sick, or too fat	17.4
Obese, or have a lot of fat	
Sleepy	
Being sick	
Feel hungry	
My body can't stand it.	
Tired	
Lazy	
No energy	
Having nobody to go with	6.5
My parents don't have time to take me to do physical activity	
Nobody go with me	
Myself	
Disapproval from others	5.2
My parents don't agree	
I, myself don't approve	
Classmates don't agree	
School don't agree	
Not being willing	5.2
Don't want to do physical activity	
Lazy	
Don't want to	
Being in a bad mood	5.1
Don't have mood	
Teacher criticize me, so that I don't have a good mood	
Bad mood	
Having no court or facility	5.1
Don't have facility	
Don't have court	
Society is messy	
More car in cities	
Grounds	
Insufficiency of facility	
Having TV, computer games, and other things to do instead	4.5
Good game to play	
Play computer	
Watching TV	
Have good movie	
Having activities that are not fun or too hard	3.2
Activity is too vigorous	
Don't have fun activity to play	
Boring activity	

## Discussion

While a number of the psychosocial factors underlying the decision to engage in physical activity identified among these middle school students from Beijing seem to be similar to those found with students in England and the United States [26-28], there are some clear differences. The physical health advantages of feeling healthy, staying in shape, and controlling weight were perceived by the students in the studies from England, the United States, and China. Similarly, the disadvantages of being tired or getting injured were identified in common. Two of the studies, this study and the study of adolescents in the United States [27], elicited easy and difficult circumstances. In both, students mentioned time, motivation, support, and access to facilities as barriers and facilitators. However, several beliefs about the relationship between participating in physical activity and academics seemed to be unique to this sample of middle school students from Beijing. More specifically, "having energy to study" and "improving my grades in physical education" were mentioned as advantages; "taking time away from studying" was mentioned as a disadvantage; and "having fewer assignments" and "having too many assignments" were mentioned as easy and difficult circumstances. In terms of salient social referents, family members were important for these students as well as for the samples of students from England [26] and the US [27]. Not surprising given the one child policy in China, siblings were not mentioned as people who would approve or disapprove in this study of middle school students from Beijing. While more rigorous quantitative research with larger samples is necessary to explore these unique beliefs, these qualitative findings suggests that some of the underlying beliefs found in other populations might not be applicable to the Beijing students and confirms the recommendation to conduct salient belief elicitations in the specific priority group of interest as a first step in formative research to design interventions.

**Figure 1:**
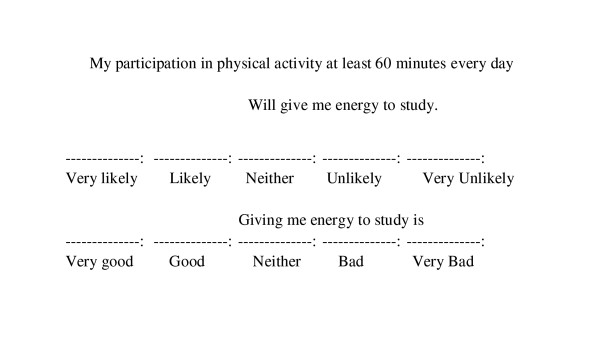
Sample Close-ended Items for Salient Consequences.

This type of rapid elicitation method to identify the salient consequences, referents, and circumstances of the behavior to be changed with the members of the population of interest can serve two major purposes [24]. Elicitation results are essential to designing close-ended items for theory-based instruments to be used in larger scale quantitative formative research to identify these psychosocial determinants. Results in Chinese are available on request. Following the recommendations of the TPB [17,18], the salient consequences, referents, and circumstances presented in Tables 3 through 8 can be used to generate measures of behavioral beliefs, normative beliefs, and control beliefs. For example, two close-ended items can be created for the salient consequences presented in Tables 4 and 5: one item would assess the strength of the participant's belief that performing the behavior would lead to the consequence; and the other would assess how positive or negative the consequence is perceived to be. For the consequence, "will give me energy to study", the two items in a self-completion format would be (figure 1)

In addition, the results of the qualitative study can suggest implications for interventions to increase physical activity among middle school students in Beijing and to provide program planners terminology and words in the language of the population of interest to use in these interventions. Again, while they will need to be confirmed by quantitative research with a larger sample, three related implications might be explored.

First, according to this study, these Chinese middle school students perceived not having enough time and having too many assignments as barriers to daily participation in physical activity. Clearly, these perceptions are consistent with their environment. The Chinese youth are under great pressure to perform well in school and heavy homework loads are typical [30]. Under the parental and societal pressures for academic success manifested in the Chinese education policy changes [5], the Chinese students tend to spend all the time studying after school. A study in Jiangsu province indicated adolescents from 12 to 14 years of age in junior high schools spent 10.8 hours per day on studies (in school and at home) during weekdays [31]. Participation in moderate to vigorous physical activity outside of school among Chinese adolescents is almost non-existent [5]. A study of environmental factors and physical activity [32] in Xi'an city found lack of extracurricular exercise in school and fewer sports meetings were associated with physical inactivity. While promoting the benefits of physical activity to physical health is one approach to increasing physical activity, these data suggest that the issue of physical activity and academic achievement must be also be addressed in prevention interventions. Many students perceived other benefits to participating in physical activity, including increasing energy to study, being fun, and being relaxing. To be effective for Beijing middle school students, an intervention encouraging physical activity might need to address the relationship between physical activity and academic success and achievement.

Second, given these findings and the one child policy in China, the composition of the social referents for middle school students is relatively simple. Parents, especially the mother, are the main sources of approval and disapproval for engaging in physical activity. To be successful, prevention programs that help middle school students increase their physical activity will likely need to deal with the perceived social pressure from family members, particularly from parents. Teachers are also important as sources of approval and disapproval. The school and home are the major social environments for the middle school students. Hence, these data suggest that interventions to encourage physical activity should engage the family and the school.

Third, these findings suggest that prevention interventions address the students' environment. Clearly, the factors of "not having enough time" and "having too many assignments" are factors that are not under the control of most middle school students. While education and communication with students will be important components of prevention interventions encouraging physical activity, to be effective, it is likely that these interventions will need to address the issue of physical activity and academic achievement not just as intrapersonal and individual factors but also as social and environmental factors. Further, given the role of parents and teachers as sources of approval, it will be important to determine the views of these groups when it comes to physical activity and academic achievement. Finally, the findings on access to facilities perceived by these students confirms a study in Xi'an city of China which found that access to community recreational facilities and concerns about safety in the neighborhood were associated with inactivity [30].

Whatever approach is taken to increasing physical activity, policy approaches need to be explored to build supportive school environment. In 1995, the World Health Organization launched the "Global School Health Initiative" which offers a vision for how to develop a school that promotes the health of its students [33]. The experience of developing Health Promoting Schools in Zhejiang province [34], Fujian province [35], Hong Kong [36] and Taiwan [37] all demonstrated the positive changes of school setting, involvement of family, positive behavior changes and learning potential among Chinese students. Therefore, a model of Health Promoting School could be a good approach to promote physical activity. The study has limitations. It is a qualitative study that is meant to provide information useful for a more rigorous and larger scale quantitative study. Thus, the data are responses to open-ended questions and the opportunity for quantitative comparisons is limited. The sample size is small and based on a convenience sample from one school district in Beijing. It examined the perceptions of physical activity behavior held by only middle school students. Thus, the results from this study are more suggestive than affirmative.

## Conclusion

The results of this qualitative study suggest that student perceptions of the relationship between their participation in physical activity and their school work might be more important factors underlying their decisions to participate in physical activity than their beliefs about the health benefits. Programs to increase physical activity should address the social and environmental factors underlying these perceptions with the goal of strengthening student engagement in the physical activity and improving learning potentials. Quantitative studies with a larger and representative sample and with close-ended items based on the qualitative research are needed to more fully understand middle school students' decisions to engage in daily physical activity. In addition, given the role of the parents as a social referent and the students' perception of the connection between physical activity and academic performance, research is needed to understand the views of parents, teachers, and school administrators.

## Competing interests

The author(s) declare that they have no competing interests.

## Authors' contributions

JZ drafted the manuscript, coordinated and participated in the study design, translated the open-ended data, and conducted the content and cross-tabulation analysis.

SEM provided oversight in the design of the elicitation, supervised the content analysis and cross-tabulation analysis, and revised the manuscript.

CYJ directed the study design and coordinated this study.

All the authors read and approved the final manuscript.
